# AKI and Mortality in Children Hospitalized With Malnutrition

**DOI:** 10.1016/j.ekir.2026.106564

**Published:** 2026-04-22

**Authors:** Anthony Batte, Aidah Namugumya, Denise C. Hasson, Aline Desmarais, Michael J. Goings, Arthur Mpimbaza, Harriet M. Babikako, Quique Bassat, Andrea L. Conroy

**Affiliations:** 1 Child Health and Development Center, College of Health Sciences, Makerere University, Kampala, Uganda; 2 Facultat de Medicina i Ciències de la Salut, Universitat de Barcelona, Barcelona, Spain; 3 Global Health Uganda, Kampala, Uganda; 4 Department of Paediatrics, College of Health Sciences, Makerere University, Kampala, Uganda; 5 Hassenfeld Children’s Hospital at NYU Langone Health, New York, New York, USA; 6 Ryan White Center for Pediatric Infectious Disease and Global Health, Indiana University School of Medicine, Indianapolis, Indiana, USA; 7 Child and Family Foundation Uganda, Kampala, Uganda; 8 ISGlobal, Hospital Clínic - Universitat de Barcelona, Barcelona, Spain; 9 Centro de Investigação em Saúde de Manhiça, Maputo, Mozambique; 10 ICREA, Pg. Lluís Companys 23, Barcelona, Spain

**Keywords:** acute kidney injury, acute malnutrition, children, definitions, mortality, risk score

## Abstract

**Introduction:**

Acute kidney injury (AKI) is a common complication of pediatric hospitalizations. We evaluated the prevalence of AKI in children hospitalized with acute malnutrition, examined how AKI definitions influence risk stratification, and assessed the performance of the STOP AKI risk score.

**Methods:**

We enrolled 185 Ugandan children hospitalized with acute malnutrition. AKI was defined using the Kidney Disease: Improving Global Outcomes (KDIGO) criteria based on serial creatinine measurements, with the nadir creatinine during hospitalization as baseline. Because children with malnutrition have low baseline creatinine levels, we evaluated whether applying minimum absolute creatinine thresholds improved identification of clinically meaningful AKI, defined by its association with mortality.

**Results:**

The median age was 1.2 years, and 13.5% of children died. KDIGO-defined AKI without a minimum creatinine threshold was not associated with mortality. Applying a minimum absolute creatinine threshold of 0.4 mg/dl to the KDIGO criteria identified AKI in 23.2% of participants and was independently associated with mortality (adjusted odds ratio [OR, aOR]: 4.06, 95% confidence interval [CI]: 1.39–11.84). A threshold of 0.5 mg/dl identified fewer children but predominantly severe AKI. Clinical risk factors included diarrhea, vomiting, and sepsis. The ISN STOP AKI risk score did not discriminate AKI risk (area under the receiver operating characteristic curve [AUROC]: 0.50, 95% CI: 0.40–0.60).

**Conclusion:**

AKI is common in children hospitalized with acute malnutrition and is strongly associated with mortality when clinically meaningful creatinine thresholds are applied. Existing AKI risk tools perform poorly in this population, thereby underscoring the need for adapted approaches to AKI identification in malnourished children.

## Graphical abstract

**Figure:**
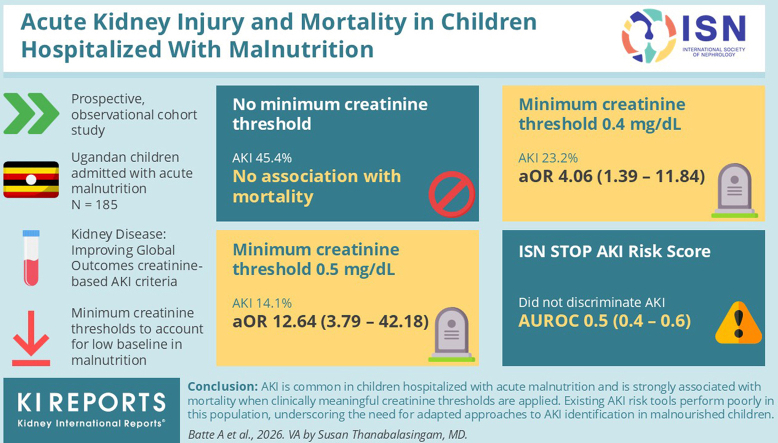

Childhood malnutrition, including acute (wasting) and chronic (stunting) forms, remains a major global health challenge. In 2022, 45 million children aged < 5 years worldwide experienced acute malnutrition, with 27% living in Africa.^1^ Malnutrition arises from complex interactions between food insecurity, socioeconomic disadvantage, chronic disease, and inflammation; and substantially increases susceptibility to infection, hospitalization, and death.^2^ Although AKI complicates an estimated one-third of pediatric hospitalizations globally,^3^ data describing AKI among children hospitalized with malnutrition are limited.

AKI is defined as an abrupt loss of kidney function and is operationally assessed using increases in serum creatinine or decreases in urine output.^4^ Standardized AKI definitions have substantially advanced epidemiologic understanding of AKI and enabled risk stratification across diverse settings.^5^ However, accurate identification of AKI depends on reliable baseline kidney function and adequate analytical precision in the measurement of serum creatinine. In children with acute malnutrition, markedly reduced muscle mass results in very low baseline serum creatinine concentrations, which may complicate the interpretation of relative creatinine changes and lead to misclassification of AKI.^6^ In high-resource settings, minimum absolute serum creatinine thresholds (commonly 0.5 mg/dl) have been applied when using the KDIGO criteria to reduce spurious AKI classification at low creatinine levels.^7^, ^8^, ^9^, ^10^, ^11^ Whether such thresholds are appropriate in children with acute malnutrition, who may have even lower baseline creatinine values, remains unclear.

Malnutrition contributes to 40% to 45% of childhood deaths globally,^12^^,^^13^ and mortality among children hospitalized with severe acute malnutrition remains high, ranging from 20% to 30%.^12^^,^^14^ AKI is an established independent predictor of mortality among hospitalized children, with the majority of AKI-related deaths occurring in low-and middle-income countries (LMICs).^15^^,^^16^ Despite the recognition that malnutrition increases vulnerability to kidney injury, most evidence is derived from adult populations; pediatric data from LMICs, where the burden of malnutrition is greatest, are scarce. Importantly, if standard creatinine-based AKI definitions perform poorly in this population, misclassification may obscure the true risk and limit opportunities for targeted intervention.

In this prospective study of Ugandan children hospitalized with acute malnutrition, we evaluated how alternative approaches to defining creatinine-based AKI influence risk stratification, using in-hospital mortality as a clinically meaningful benchmark. Specifically, we assessed whether applying minimum absolute serum creatinine thresholds to the KDIGO criteria improves identification of AKI associated with mortality. We further evaluated the performance of the ISN STOP AKI risk score in this high-risk pediatric population.

## Methods

### Study Design

This prospective observational cohort study enrolled 185 children hospitalized with acute malnutrition at Mulago National Referral Hospital in Kampala, Uganda, between September 2020 and February 2021. Children aged 6 months to 10 years were eligible if they were hospitalized for acute malnutrition, defined as wasting (weight-for-height) or underweight (weight-for-age) z scores ≤ −2 using the World Health Organization 2007 anthropometry reference standards.^17^^,^^18^ Participants were excluded if they were unwilling to complete study procedures.

All children in the study were managed according to Uganda guidelines for acute malnutrition.^19^ Hospitalized children routinely receive empiric antibiotics, with 85% of children receiving amikacin during the study period. Typically, as per Uganda National Treatment Guidelines, children hospitalized with acute malnutrition are treated with ampicillin and gentamicin. However, because of Ministry of Health advisories regarding gentamicin-associated adverse events between 2018 and 2020, alternative aminoglycosides were commonly used.

To estimate sample size, we assumed 80% power, an alpha of 0.05, and an AKI prevalence of 26% among hospitalized children, with AKI-associated mortality increasing from 4% to 18%.^20^ The calculated sample size was 175, increased to 185 to account for potential loss to follow-up.

### Study Procedures

On the day of admission, each enrolled child underwent a standardized clinical assessment, including medical history and a physical examination conducted by a study doctor. Laboratory investigations at enrolment included complete blood count, peripheral blood smear to assess for malaria, renal function tests, and urinalysis. Malaria was assessed using Giemsa-stained peripheral blood smears, read independently by 2 microscopists, with a third reader adjudicating discrepant results, and by rapid diagnostic testing (Malaria Ag P.f/Pan, Abbott). A spot urine sample was collected for dipstick urinalysis, microscopy, and measurement of urine albumin and creatinine.

### Study Definitions

#### Comorbid Illnesses

HIV was diagnosed and managed following the Uganda Ministry of Health guidelines testing algorithm where the point-of-care antibody test (Alere Determine) was used for screening, Stat-Pak for confirmation, and SD Bioline as a tie-breaker test. For children aged < 18 months, HIV infection was confirmed using DNA PCR testing.^21^ Tuberculosis was diagnosed in accordance with national guidelines using microbiologic, radiographic, or clinical criteria consistent with tuberculosis in malnourished children (full details in the Supplementary Methods). Sepsis was defined using the international consensus criteria as abnormal temperature (> 38.5 °C or < 36 °C), or age-specific abnormal leukocyte count (leukocytosis or leukopenia) in the presence of either age-specific tachycardia or tachypnea, or systolic blood pressure < 5th percentile for age.^22^ Acute infection was defined as having sepsis or malaria. Anemia was defined using the World Health Organization age-specific hemoglobin thresholds: < 10.5 g/dl (6 months to <2 years), < 11.0 g/dl (2 to < 5 years), and < 11.5 g/dl (> 5 years).^23^

#### Assessment of AKI

AKI was defined using the KDIGO consensus criteria based on changes in serum creatinine.^4^ AKI was defined as a ≥1.5-fold increase in creatinine from baseline within 7 days or an absolute increase of ≥0.3 mg/dl within 48 hours. AKI staging followed the KDIGO guidelines as follows: stage 1 (1.5- to 1.9-fold increase from baseline or ≥ 0.3 mg/dl increase within 48 hours), stage 2 (2.0- to 2.9-fold increase), and stage 3 (>3.0-fold increase, creatinine ≥ 4 mg/dl, an estimated glomerular filtration rate < 35 ml/min per 1.73 m^2^, or indication for dialysis).^4^ Serum creatinine was measured at enrolment (at admission to hospital), within 48 hours, on day 7, and at discharge. Creatinine was measured using a Cobas analyzer by the modified Jaffe method, with a lower detection limit of 0.17 mg/dl (15 μmol/l). Values below the assay detection limit were assigned a value of 0.16 mg/dl.

Because preillness creatinine values were unavailable, the lowest creatinine measured during hospitalization was used as the baseline value for primary analyses. Eighteen participants had only a single creatinine measurement due to early discharge (*n* = 6), death before 48-hour sampling (*n* = 10), or missing serial samples (*n* = 2). For those children, baseline creatinine was estimated using a height-independent approach assuming a normal glomerular filtration rate of 120 ml/min per 1.73 m^2^.^24^^,^^25^ Participant characteristics comparing nadir-based and estimated baseline approaches are presented in Supplementary Table S1.

To identify AKI definitions associated with clinically meaningful increases in mortality, we evaluated the frequency of AKI and its relationship with mortality using within-patient changes in creatinine using the KDIGO criteria and applied minimum absolute serum creatinine thresholds (definition 1, no minimum creatinine threshold; definition 2, minimum absolute creatinine threshold of 0.4 mg/dl; definition 3, minimum absolute creatinine threshold of 0.5 mg/dl). Under threshold-based definitions, the peak creatinine during hospitalization was required to exceed the specified threshold in addition to meeting the KDIGO criteria. For example, an increase from 0.2 mg/dl to a peak of 0.3 mg/dl (≥ 1.5-fold increase) meets the KDIGO criteria but would not be classified as AKI because the peak creatinine did not exceed 0.4 mg/dl or 0.5 mg/dl. In secondary analyses, baseline creatinine was estimated for all participants as described and AKI defined based on serial creatinine measurements or admission creatinine alone, reflecting settings where serial measurements may not be available.

### AKI Risk Score for Children

The ISN 0by25 initiative promotes the 5R framework (Risk assessment, Recognition, Response, Renal support, and Rehabilitation) for AKI prevention and management.^26^ As part of the risk assessment component, the ISN developed a clinical AKI risk score assigning weighted points to predefined clinical features, including history of kidney disease, oliguria, infection with fever, hypotension or shock, pregnancy with hypertension or seizures, body swelling, loss of appetite, HIV on antiretroviral therapy, altered mental status (coma/confusion), and anemia or pallor.^27^ In this study, we evaluated the performance of the STOP AKI risk score in predicting KDIGO-defined AKI. Clinical variables were operationalized using standardized definitions. Respiratory infection was defined as cough and tachypnea with chest indrawing or stridor.^28^ Shock in children with acute malnutrition was defined as lethargy or unconsciousness with cold extremities and either prolonged capillary refill (> 3 seconds), tachycardia, or weak pulse.^19^ Inability to breastfeed or drink was used to define loss of appetite. Coma was defined using the Blantyre coma scale of <3, and confusion as an inappropriate verbal response on neurological exam. The risk scores generated were categorized as low risk (0–2), moderate (3–5), or high (> 6) risk of AKI.^27^

### Data Analysis

Data analysis was conducted using STATA v18.0 (StataCorp). Categorical data were summarized as frequencies and percentages whereas continuous data were summarized using medians with interquartile ranges. The Chi-square test or Fisher exact test were used to evaluate differences between categorical variables and AKI, as appropriate. The Wilcoxon rank-sum test was used to assess the differences between continuous variables and AKI. Logistic regression was used to evaluate the risk factors for AKI estimating unadjusted ORs and aORs. In bivariate analysis, factors with a *P*-value < 0.2 were added to the multivariate logistic regression models. Age and parent reported biological sex were added to all multivariate logistic regression models. At multivariate logistic regression analysis, a *P*-value < 0.05 was considered significant. Model calibration was evaluated using the Hosmer-Lemeshow goodness of-fit test with a *P* > 0.05 indicating that the model fits the data well. Kaplan Meier curves were constructed for in-hospital mortality based on different AKI definitions. Nonparametric receiver operating characteristic curves were used to evaluate the discriminatory ability of the AKI risk score to predict AKI. A modified AKI risk score was developed by incorporating aORs for variables significantly associated with AKI, using a similar approach to that used in the original AKI score development.^27^ AUROCs were compared using the method of Delong.

### Ethical Approval

Written informed consent was obtained from all study participants' parents or legal guardians, and assent was obtained, when feasible, for children aged ≥ 8 years. This study was approved by the Institutional Review Board of Makerere University School of Biomedical Sciences Research and Ethics Committee (first approval date: January 21, 2020, IRB number SBS-733). The study was also approved by the Uganda National Council for Science and Technology (approval date: July 13, 2020, approval number; HS 2768).

## Results

### Description of Study Population

**Figure 1:**
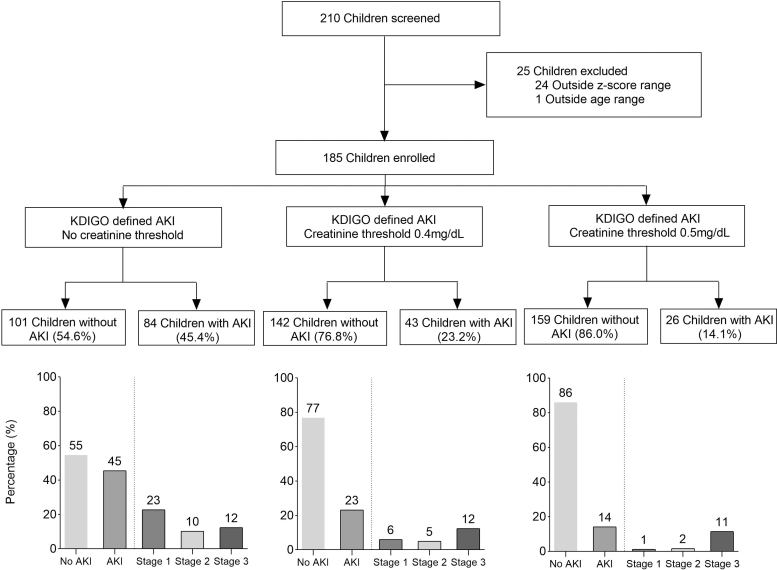
Flow chart of study population. KDIGO criteria were used to define AKI, in the first instance no creatinine threshold applied. We then added a creatinine threshold implying that a child had to have a minimum creatinine of a given threshold on addition to the KDIGO criteria to have a diagnosis of AKI. Creatinine thresholds of 0.4 mg/dl and 0.5 mg/dl were applied, and the prevalence and AKI stages for each of the approaches are provided. AKI, acute kidney injury; KDIGO, Kidney Disease: Improving Global Outcomes.

A total of 185 children hospitalized with acute malnutrition were enrolled (Figure 1). The median age was 1.2 (interquartile range: 0.9–1.7) years and 63.8% were male (Table 1). The most common presenting symptoms were diarrhea (58.4%) and vomiting (51.4%). Evidence of acute infection was frequent: 4.9% had malaria and 36.2% met clinical criteria for sepsis. Chronic comorbidities were present in 36.2% of children, including HIV infection (13.5%), tuberculosis (16.2%), and cerebral palsy and global developmental delay (10.8%) (Supplementary Figure S1). The median length of hospital stay was 15 (interquartile range: 7–23) days and 74.6% of children were hospitalized for >7 days. Overall, 13.5% of children died during hospitalization. Among the deaths, 28.0% occurred within 48 hours of admission, and 76.0% within the first 7 days.

**Table 1:** Demographic and clinical characteristics of the participants

Demographics	Study population (*N* = 185)
Age, yrs, median (IQR)	1.2 (0.9–1.7)
Age categories, *n* (%)	
≤ 1 yrs	68 (36.8)
> 1–2 yrs	81 (43.8)
> 2 yrs	36 (19.5)
Sex, *n* (%) female	67 (36.2)
Clinical characteristics on admission	
Fever, *n* (%)	63 (34.1)
Features of fluid loss	
No diarrhea or vomiting	55 (29.7)
Either diarrhea or vomiting	57 (30.8)
Both diarrhea and vomiting	73 (39.5)
Unable to drink/breastfeed, *n* (%)	35 (18.9)
Anemia, *n* (%)	120 (64.9)
Reduce urine output, *n* (%)	9 (4.9)
Comorbid conditions	
Cerebral palsy/ Global developmental delay, *n* (%)	20 (10.8)
Human immunodeficiency virus infection, *n* (%)	25 (13.5)
Tuberculosis, *n* (%)	30 (16.2)
Sepsis, *n* (%)	67 (36.2)
Malaria, *n* (%)	9 (4.9)
Acute infection^a^, *n* (%)	74 (40.0)
Chronic illness^b^, *n* (%)	67 (36.2)
Length of hospital stay (d)	15 (7–23)
Laboratory findings	
WBC × 10^3^/μl	11.8 (8.6–16.1)
Neutrophil count × 10^3^/μl	3.8 (2.3–6.8)
Lymphocyte count × 10^3^/μl	5.2 (3.7–7.7)
Hemoglobin, g/dl	9.8 (8.3–11.1)
Platelet count × 10^9^/L	389.0 (251.0–517.0)
Creatinine (mg/dl)	0.3 (0.2–0.4)
Blood urea nitrogen (BUN) (mg/dl)	5.3 (3.4–8.5)
Sodium (mmol/l)	135.2 (133.0–138.0)
Potassium (mmol/l)	3.9 (3.6–4.2)
Chloride (mmol/l)	98.0 (96.0–99.0)
Treatment during hospitalization	
Paracetamol, *n* (%)	41 (22.2)
Ampicillin, *n* (%)	146 (78.9)
Amikacin, *n* (%)	158 (85.4)
Ceftriaxone, *n* (%)	113 (61.1)
Highly active anti-retroviral therapy, *n* (%)	19 (10.3)
Anti-Tuberculosis medications, *n* (%)	30 (16.2)
Outcome	
In-hospital mortality, *n* (%)	25 (13.5)

IQR, interquartile range; WBC, white blood cell.

Data presented as median (IQR) or *n* (%).

a Acute infection is defined as malaria or sepsis.

b Chronic illness includes HIV infection, tuberculosis, cerebral palsy/global developmental delay, epilepsy, cleft palate, congenital heart disease, posterior urethral valves.

### Defining AKI and the Association With Mortality

**Figure 2:**
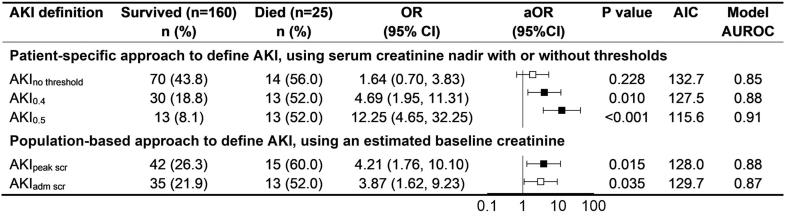
Relationship between mortality and different acute kidney injury (AKI) KDIGO definitions. The forest plot depicts the unadjusted and adjusted odd ratios showing the relationship of KDIGO defined AKI and mortality. The different AKI definitions use no serum creatinine (SCr) threshold, a minimum serum creatinine threshold of 0.4 mg/dl or a minimum serum creatinine threshold of 0.5 mg/dl. AKI_peak scr_ signifies KDIGO defined AKI using estimated baseline SCr using height-independent approach whereas AKI_adm scr_ used estimated baseline SCr but AKI defined using the single admission creatinine value. Black boxes on whisker plots indicate *P*-values < 0.05 whereas white boxes indicate *P*-values > 0.5 after adjusting for multiple comparisons. AIC, Akaike Information Criterion. aOR, adjusted odds ratio; CI, confidence interval; KDIGO, Kidney Disease: Improving Global Outcomes; OR, odds ratio.

**Figure 3:**
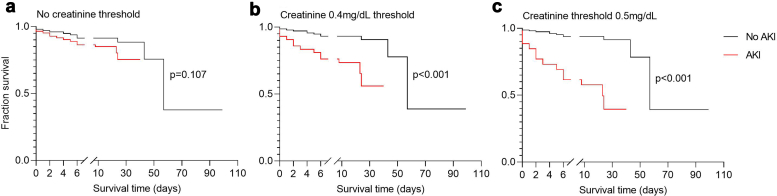
Kaplan Meier curves for mortality risk among children with and without AKI. The graphs show difference in survival of children without AKI (black line) compared with those with AKI (red line), *P*-value based on the log-rank test. (a) KDIGO-defined AKI without using a creatinine threshold is not associated with mortality while the definitions based on (b) creatinine threshold of 0.4 mg/dl and (c) 0.5mg/dl are associated with mortality. AKI, acute kidney injury.

A clinically meaningful definition of AKI should identify children at increased risk of death. Using serial creatinine measurements without a minimum creatinine threshold, 84 children (45.4%) met the KDIGO criteria for AKI. Applying a minimum absolute creatinine threshold reduced the frequency of AKI to 23.2% at ≥ 0.4 mg/dl and 14.1% at ≥ 0.5 mg/dl (Figure 1). AKI defined using minimum creatinine thresholds was associated with mortality. A threshold of ≥ 0.4 mg/dl was associated with increased odds of death (OR: 4.69, 95% CI: 1.95–11.31; *P* = 0.001), as was a threshold of ≥ 0.5 mg/dl (OR: 12.25, 95% CI: 4.65–32.25; *P* < 0.001). These associations remained significant after adjustment for covariates: ≥ 0.4 mg/dl (aOR 4.06, 95% CI: 1.39–11.84; *P* = 0.010) and ≥ 0.5 mg/dl (aOR: 12.64, 95% CI 3.79–42.18; *P* < 0.001) (Figure 2). Sensitivity and specificity for each AKI definition in predicting mortality are provided in Supplementary Table S2. Among the 25 children who died, 19 died within 7 days (76%), and 11 children who died (57.9%) met the AKI definition without creatinine threshold, and 10 (52.6%) met the AKI definition using creatinine thresholds of 0.4 mg/dl or 0.5 mg/dl. Kaplan–Meier survival analysis showed significantly lower survival among children meeting AKI definitions based on creatinine thresholds of 0.4 mg/dl and 0.5 mg/dl compared with those who did not (log-rank *P* < 0.001 for both) (Figure 3).

Defining AKI using serial creatinine measurements with a population-estimated baseline creatinine was independently associated with mortality (aOR: 3.89, 95% CI: 1.30–11.60; *P* = 0.015). In contrast, defining AKI using admission creatinine alone with an estimated baseline was not associated with mortality after multivariable adjustment (Figure 2). When comparing AKI definitions based on (i) within-patient nadir baseline with a ≥0.4 mg/dl threshold and (ii) population-estimated baseline using serial creatinine measurements, model performance was similar (Akaike information criteria, 127.5 and 128.0; AUROC of 0.88 for both approaches) (Figure 2). All models were adjusted for age, sex, diarrhea, vomiting, hepatomegaly, tuberculosis, sepsis, shock, lymphocyte count, and platelet count. Using the Hosmer-Lemeshow Goodness-of-fit test, all models were well-calibrated with the model of KDIGO-defined AKI without a creatinine threshold having a Hosmer–Lemeshow chi-square of 1.79, *P* = 0.987; the model with a creatinine threshold of 0.4mg/dl having a chi-square of 2.43, *P* = 0.965; and the model with a creatinine threshold of 0.5mg/dl having a chi-square of 4.45 and a *P* = 0.815.

### Clinical Parameters Associated With AKI

We evaluated clinical predictors of KDIGO-defined AKI using the ≥ 0.4 mg/dl minimum creatinine threshold, because this definition balanced sensitivity and specificity and was independently associated with mortality.

In multivariable analysis, diarrhea and vomiting were strongly associated with AKI (aOR: 5.43, 95% CI: 1.91–15.42; *P* = 0.001), as was sepsis (aOR: 2.18, 95% CI: 1.01–4.72; *P* = 0.047) (Table 2). AKI was not associated with a composite measure of acute infection (malaria or sepsis) (OR: 1.60, 95% CI: 0.81–3.19; *P* = 0.179) or with chronic comorbidity (HIV, tuberculosis, cerebral palsy/global developmental delay, cleft palate, congenital heart disease, posterior urethral valves, or epilepsy) (OR: 1.06, 95% CI: 0.52–2.14; *P* = 0.877).

**Table 2:** Predictors of KDIGO-defined AKI with a minimum absolute creatinine threshold of 0.4 mg/dl

Clinical characteristics	Unadjusted estimate				Adjusted estimate	
	No AKI (*n* = 142)	AKI_0.4_ (*n* = 43)	OR (95% CI)	*P* value	aOR (95% CI)	*P*-value
Age, yrs, median (interquartile range)	1.2 (0.9–1.7)	1.1 (0.7–2.1)	0.96 (0.68–1.34)	0.793	1.08 (0.72–1.61)	0.722
Sex, *n* (%) Female	50 (35.2)	17 (39.5)	1.20 (0.60–2.43)	0.606	1.36 (0.64–2.90)	0.424
Features of fluid loss						
No diarrhea or vomiting	49 (34.5)	6 (14.0)	Ref			
Either diarrhea or vomiting	44 (31.0)	13 (30.2)	2.41 (0.84–6.89)	0.100	2.61 (0.87–7.80)	0.086
Both diarrhea and vomiting	49 (34.5)	24 (55.8)	4.00 (1.50–10.64)	0.005	5.43 (1.91–15.42)	0.001
Comorbid conditions						
Cerebral palsy / global developmental delay, *n* (%)	14 (9.9)	6 (14.0)	1.48 (0.53–4.13)	0.451		
HIV infection, *n* (%)	18 (12.7)	7 (16.3)	1.34 (0.52–3.46)	0.546		
Tuberculosis, *n* (%)	24 (16.9)	6 (14.0)	0.80 (0.30–2.10)	0.646		
Sepsis, *n* (%)	46 (32.4)	21 (48.8)	1.99 (1.00–3.99)	0.051	2.18 (1.01–4.72)	0.047
Malaria, *n* (%)	9 (6.3)	0 (0.0)	---	---		
Acute infection^a^, *n* (%)	53 (37.3)	21 (48.8)	1.60 (0.81–3.19)	0.179		
Chronic illness^b^*n* (%)	51 (35.9)	16 (37.2)	1.06 (0.52–2.14)	0.877		
AKI risk score variables						
History of kidney disease, *n* (%)	0 (0.0)	1 (2.3)	---	---		
History of reduced urine output, *n* (%)	6 (4.2)	3 (7.0)	1.70 (0.41–7.10)	0.467		
Respiratory infection with fever, *n* (%)	11 (7.7)	4 (9.3)	1.22 (0.37–4.05)	0.744		
Hypotension or shock, *n* (%)	6 (4.2)	4 (9.3)	2.32 (0.62–8.65)	0.208		
Body swelling, *n* (%)	55 (38.7)	10 (23.3)	0.48 (0.22–1.05)	0.066	0.47 (0.20–1.07)	0.073
Unable to drink/breastfeed, *n* (%)	24 (16.9)	11 (25.6)	1.69 (0.75–3.81)	0.206		
HIV on highly active antiretroviral therapy, *n* (%)	14 (9.9)	5 (11.6)	1.20 (0.41–3.55)	0.738		
Coma/ confusion, *n* (%)	11 (7.7)	6 (14.0)	1.93 (0.67–5.57)	0.223		
Anemia, *n* (%)	95 (66.9)	25 (58.1)	0.69 (0.34–1.38)	0.293		
Medications during hospitalization						
Paracetamol, *n* (%)	28 (19.7)	13 (30.2)	1.76 (0.82–3.81)	0.149	1.48 (0.63–3.49)	0.371
Ampicillin, *n* (%)	115 (81.0)	31 (72.1)	0.61 (0.28–1.33)	0.213		
Amikacin, *n* (%)	121 (85.2)	37 (86.0)	1.07 (0.40–2.85)	0.892		
Ceftriaxone, *n* (%)	84 (59.2)	29 (67.4)	1.43 (0.70–2.94)	0.330		
Anti-tuberculosis medications, *n* (%)	24 (16.9)	6 (14.0)	0.80 (0.30–2.10)	0.646		
Laboratory findings, median (interquartile range)						
WBC count × 10^3^/μl^c^	11.1 (8.6–14.9)	14.2 (8.6–20.8)	1.05 (1.01–1.09)	0.008		
Neutrophil count × 10^3^/μl	3.4 (2.2–5.2)	6.0 (2.7–12.2)	1.10 (1.04–1.16)	0.001		
Lymphocyte count × 10^3^/μl	5.4 (3.9–7.7)	5.0 (3.2–7.9)	0.97 (0.82–1.12)	0.587		
Hemoglobin, g/dl	9.9 (8.4–11.1)	9.6 (7.8–11.1)	0.96 (0.82–1.12)	0.595		
Platelet count × 10^3^/μl	388 (269–520)	408 (228–514)	1.00 (1.00–1.00)	0.587		

AKI, acute kidney injury; aOR, adjusted OR; CI, confidence interval; KDIGO, Kidney Disease: Improving Global Outcomes; OR, odds ratio; WBC, white blood cell.

a Acute infection is defined as malaria or sepsis.

b Chronic illness includes HIV infection, tuberculosis, cerebral palsy/global developmental delay, epilepsy, cleft palate, congenital heart disease, posterior urethral valves.

c Because WBC counts were used in defining sepsis, WBC parameters were not added to the final adjusted model to limit collinearity.

In bivariate analyses, clinical predictors of KDIGO-defined AKI were similar when applying either a ≥0.4 mg/dl or ≥ 0.5 mg/dl threshold (Supplementary Table S3). Participant clinical characteristics, laboratory findings, AKI frequency, and mortality did not differ by sex (Supplementary Table S4).

### Comparison of Risk Scores in Predicting AKI

**Figure 4:**
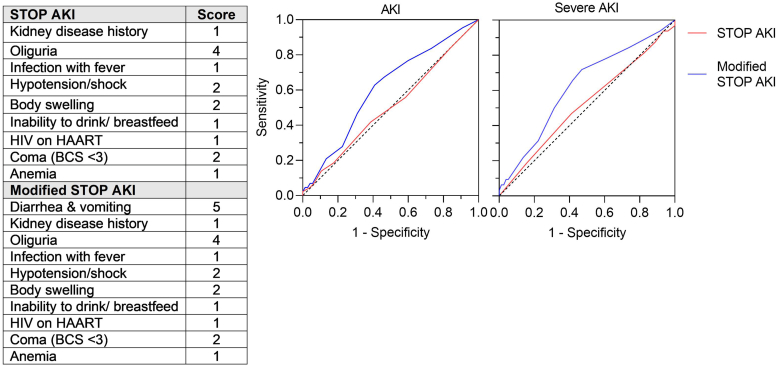
Receiver operating characteristic curves for acute kidney injury (AKI) risk score prediction of AKI. The graph shows the different receiver operator curves for the STOP AKI score (red line), and the modified STOP AKI risk score (blue line) in predicting AKI and severe AKI. The table shows the different variables used for each score. BCS, Blantyre coma scale; HAART, highly active antiretroviral therapy.

The ISN STOP AKI risk score was developed to identify patients at increased risk of AKI (Figure 4). Using this score, 39.5% of children had a score ≥ 3, corresponding to moderate-to-high risk. The STOP AKI score demonstrated poor discrimination for KDIGO-defined AKI, with an AUROC of 0.50 (95% CI: 0.40–0.60), sensitivity of 63.2% (95% CI: 52.5–73.9), and specificity of 39.0% (95% CI: 28.2–49.8). Discrimination was similarly limited for severe AKI (stage 2–3; AUROC: 0.48, 95% CI: 0.37–0.59) and for the composite outcome of death or severe AKI (AUROC: 0.53, 95% CI: 0.43–0.62) (Table 3). Given the limited performance of the STOP AKI score in this population, we evaluated a modified STOP AKI score incorporating diarrhea and vomiting. The modified score demonstrated modest improvement in discrimination for AKI (AUROC: 0.61, 95% CI: 0.51–0.70), severe AKI (AUROC: 0.62, 95% CI: 0.51–0.73), and the composite outcome of death or severe AKI (AUROC: 0.64, 95% CI: 0.55–0.74) (Figure 4). The modified score significantly improved discrimination for AKI and severe AKI compared with the original score (*P* = 0.041 and *P* = 0.013, respectively) (Table 3).

**Table 3:** Performance of the AKI risk score in predicting AKI and severe AKI

Measure	AKI_04_ AUROC (95% CI)	Severe AKI_04_ AUROC (95% CI)	Composite (severe AKI, death)
AKI risk score	0.50 (0.40–0.60)	0.48 (0.37–0.59)	0.53 (0.43–0.62)
Modified AKI risk score	0.61 (0.51–0.70)	0.62 (0.51–0.73)	0.64 (0.55–0.74)
*P*-value comparing scores	0.041	0.013	0.015

AKI, acute kidney injury; AUROC, area under the receiver operating characteristic curve; CI, confidence interval.

## Discussion

Children in LMICs are frequently hospitalized with acute malnutrition, yet data describing the frequency of AKI and the prognostic significance of AKI in this population remain limited. In this study, we demonstrate that AKI occurred in 23% of children hospitalized with acute malnutrition and was independently associated with mortality. AKI was observed across children with both acute and chronic comorbidities and in those with and without documented infections. An AKI risk score developed for use in predominantly adult populations in LMICs demonstrated poor discrimination in this pediatric cohort. These findings underscore the need to adapt and validate AKI risk stratification tools for vulnerable pediatric populations.

Children with acute malnutrition have markedly reduced muscle mass and low baseline serum creatinine concentrations, increasing susceptibility to assay imprecision and potential misclassification of AKI.^29^^,^^30^ High-income settings have recommended applying a minimum absolute creatinine threshold of 0.5 mg/dl to the KDIGO definition.^7^, ^8^, ^9^, ^10^, ^11^^,^^31^ However, this threshold may be overly restrictive for children with malnutrition. When applying a threshold of 0.5 mg/dl, 92.3% of AKI identified was classified as severe (defined as AKI stage 2 or 3) compared with 51.2% severe AKI when applying a 0.4 mg/dl threshold. Although both thresholds were associated with mortality, the higher threshold identified a smaller subset of children at markedly elevated risk (aOR: 12.01, 95% CI: 3.74–38.62). Collectively, these findings suggest that a minimum creatinine threshold of 0.4 mg/dl may better balance sensitivity and clinical relevance in malnourished children by identifying AKI associated with mortality while limiting misclassification at very low creatinine values.

As part of the ISN 0by25 initiative to eliminate preventable deaths from AKI, the STOP AKI protocol was developed to facilitate risk assessment, early recognition, and management of AKI in LMICs.^26^ The associated clinical risk score has been used as a screening tool in primarily adult populations in Nepal, Malawi, and Bolivia.^27^ In this pediatric cohort of children hospitalized with acute malnutrition; however, the STOP AKI score demonstrated poor discrimination for AKI. Incorporating diarrhea and vomiting modestly improved predictive performance of the score, but overall discrimination remained limited. These findings suggest that risk stratification tools developed in adult populations may not be generalized to malnourished children, in whom the pathophysiology and clinical drivers of AKI differ. Development and validation of pediatric-specific AKI risk tools are needed to support early identification and targeted interventions in this high-risk population.

Risk factors for AKI in children with acute malnutrition are not well-established. Vomiting and diarrhea are common clinical presentations and frequent reasons for admission in this population.^32^, ^33^, ^34^ Consistent with previous studies, children with vomiting and diarrhea were more likely to develop AKI, suggesting that hypoperfusion from gastrointestinal fluid losses contributes to AKI in these children.^35^, ^36^, ^37^ Even though hypovolemia is known to contribute to AKI, fluid resuscitation is a challenge in children with acute malnutrition. The World Health Organization guidelines recommend conservative fluid management, prioritizing oral or nasogastric fluids for the treatment of acute malnutrition. I.V. fluids are restricted to children with shock or those who cannot tolerate oral fluids.^19^^,^^38^^,^^39^ The findings that gastrointestinal fluid losses are a risk factor for AKI in acute malnutrition emphasize the need to evaluate appropriate fluid replacement strategies to limit the risk of AKI due to hypoperfusion.

Children with acute malnutrition have impaired immunity and are at increased risk of infections and sepsis.^40^ In the present study, sepsis was independently associated with AKI. Sepsis-associated AKI is driven by multiple mechanisms, including inflammation, complement activation, renin-aldosterone-angiotensin system dysregulation, mitochondrial, metabolic, and circulatory dysfunction.^41^ Previous work in Ugandan children hospitalized with acute infections demonstrated systemic perturbations in inflammatory pathways in children with acute malnutrition, including elevated soluble triggering receptor expressed on myeloid cell-1 and chitinase-3-like-protein-1, and reduced C-X-C motif chemokine ligand 10, which were mediators of mortality in malnutrition.^2^ Notably, both soluble triggering receptor expressed on myeloid cell-1 and chitinase-3-like-protein-1 have been identified as biomarkers of AKI.^42^^,^^43^ Additional studies are needed to understand the complex interplay between malnutrition, infection, and AKI.

In our study, the case fatality rate was 13.5%, consistent with previous studies among hospitalized children with acute malnutrition (9.8 to 20.7%).^33^^,^^44^^,^^45^ AKI was an independent predictor of mortality, and nearly half of children with stage 3 AKI died. These findings reinforce the well-established association between AKI and mortality across diverse geographic and economic settings.^3^ However, accurate identification of creatinine-defined AKI in malnourished children remains challenging because of low muscle mass and very low baseline creatinine concentrations.^7^, ^8^, ^9^, ^10^, ^11^^,^^31^ By applying a minimum creatinine threshold of 0.4 mg/dl, we aimed to reduce misclassification while preserving detection of AKI associated with mortality. In general, even though serial changes in creatinine are a recognized approach in diagnosis of AKI, a number of limitations to its use persist, such as delay in onset of creatinine increase relative to the timing of kidney injury, the effect of muscle mass and hydration status on serum creatinine concentration.^46^^,^^47^ Further studies are thus needed to evaluate alternative biomarkers for AKI diagnosis and to elucidate the different phenotypes of AKI in this biologically complex population of children with malnutrition.

A number of limitations should be considered. Preillness creatinine values were unavailable; therefore, the nadir creatinine during hospitalization was used as the baseline estimate, except in 18 children with a single measurement, for whom baseline creatinine was estimated using a height-independent approach. Further, for children who died early or those who never achieved complete kidney function recovery, their creatinine nadir in hospital may not have declined enough to approximate their actual baseline. Given the conservative fluid management strategy in children with acute malnutrition, substantial hemodilution of creatinine is unlikely. Although KDIGO definitions incorporate urine output criteria, accurate measurement of urine output without indwelling catheterization is challenging, particularly in clinically unstable children with diarrhea. Because serial creatinine measurements were obtained during the first week of hospitalization, our analyses primarily captured community-acquired and early hospital-acquired AKI. Late hospital-acquired AKI may have been missed especially in the context of prolonged hospitalization and exposure to potentially nephrotoxic antimicrobial therapy. Finally, in defining AKI, mortality is a high specificity but low sensitivity anchor for clinically meaningful AKI, and this was a single-center study and external validation is required. Nonetheless, the cohort provides prospective data from a population of children in LMICs who are substantially underrepresented in the global AKI literature.

This study has multiple strengths. AKI was prospectively evaluated using serial creatinine measures in children hospitalized with malnutrition in a low-resource setting, addressing a major gap in the global pediatric AKI literature. Serial assessment of creatinine trajectories allowed more accurate characterization of AKI in a population with low baseline creatinine concentrations. Finally, we examined the performance of the ISN STOP AKI risk score in this high-risk pediatric cohort, providing data to inform refinement and adaptation of AKI risk stratification tools for malnourished children.

This study reinforces the importance of AKI in the triage and risk stratification of children hospitalized with acute malnutrition in LMICs. We demonstrate that applying a minimum creatinine threshold to KDIGO criteria improves identification of AKI associated with mortality in this vulnerable population. These findings have implications for clinical risk assessment and for the design of AKI surveillance and intervention strategies in malnourished children. Future research should further define the pathophysiology and phenotypes of AKI in acute malnutrition and evaluate biomarkers that complement creatinine-based definitions to improve early detection and risk stratification.

## Conclusion

AKI occurs in nearly a quarter of children hospitalized with acute malnutrition, and despite low creatinine values, is strongly associated with mortality. Clinicians should maintain a high index of suspicion for AKI in this population, particularly in children presenting with diarrhea, vomiting, or sepsis. There is a need for targeted interventions to prevent and manage AKI in children with acute malnutrition to improve outcomes. Future AKI trials and surveillance programs in malnourished children should incorporate creatinine thresholds that reflect biological and analytical constraints in this population.

## Disclosure

All the authors declared no competing interests.

## Supplementary Material

Supplementary File (PDF) **Supplemental Methods. Figure S1.** Frequency of chronic illnesses among children hospitalized with acute malnutrition. **Table S1.** Comparison of participant characteristics with single value vs. serial creatinine values. **Table S2.** Sensitivity and specificity of different AKI definitions to predict mortality. **Table S3.** Comparison of participant characteristics across different AKI definitions (modifying absolute creatinine thresholds). **Table S4.** Sex disaggregated presentation of clinical characteristics, AKI, and mortality. STROBE Statement—checklist of items that should be included in reports of observational studies.

## Footnotes

Supplementary File (PDF)

**Supplemental Methods.**
**Figure S1.** Frequency of chronic illnesses among children hospitalized with acute malnutrition. **Table S1.** Comparison of participant characteristics with single value vs. serial creatinine values. **Table S2.** Sensitivity and specificity of different AKI definitions to predict mortality. **Table S3.** Comparison of participant characteristics across different AKI definitions (modifying absolute creatinine thresholds). **Table S4.** Sex disaggregated presentation of clinical characteristics, AKI, and mortality. STROBE Statement—checklist of items that should be included in reports of observational studies.
