# Supplementary material for: AKI and Mortality in Children Hospitalized With Malnutrition
Source: Kidney Int Rep. 2026 Apr 22;11(7):106564. doi: 10.1016/j.ekir.2026.106564 (PMC13227193; doi:10.1016/j.ekir.2026.106564)
Supplement: Supplementary File (PDF) — Supplemental Methods. Figure S1. Frequency of chronic illnesses among children hospitalized with acute malnutrition. Table S1. Comparison of participant characteristics with single value vs. serial creatinine values. Table S2. Sensitivity and specificity of different AKI definitions to predict mortality. Table S3. Comparison of participant characteristics across different AKI definitions (modifying absolute creatinine thresholds). Table S4. Sex disaggregated presentation of clinical characteristics, AKI, and mortality. STROBE Statement—checklist of items that should be included in reports of observational studies. [file mmc1.pdf]

Supplemental Material for:

Batte et al., (2026) Acute kidney injury and mortality in children hospitalized with acute malnutrition submitted to *Kidney International Reports.*

Table of Contents

1. Supplementary Methods
2. Supplementary Figure S1. Frequency of chronic illnesses among children hospitalized with acute malnutrition.
3. Supplementary Table S1. Comparison of participant characteristics with single value vs. serial creatinine values
4. Supplementary Table S2: Sensitivity and specificity of different AKI definitions to predict mortality
5. Supplementary Table S3: Comparison of participant characteristics across different AKI definitions (modifying absolute creatinine thresholds)
6. Supplementary Table S4: Sex disaggregated presentation of clinical characteristics, AKI and mortality
7. STROBE Statement—checklist of items that should be included in reports of observational studies

Supplementary Methods

Study Definitions

Tuberculosis was diagnosed in 30 children as part of the clinical management using a combination of diagnostics and clinical symptoms supportive of TB in a child with malnutrition, including a chest x-ray suggestive of pulmonary TB (25/30, 83.3%), sputum positive for acid and alcohol fast bacilli (AAFB) (5/30, 16.7%), gastric lavage positive for AAFBs (1/30, 3.3%), or positive urine TB lipoarabinomannan (LAM) (1/30, 3.3%). Clinical signs included cough for more than 2 weeks (19/30, 63.3%), persistent fevers (11/30, 36.7%), failure to respond to antibiotics treatment for more than 2 weeks (10/30, 33.3%), or a history of contact with a caretaker with TB (3/30, 10%). Three children (10%) were already on TB treatment at the time of hospitalization.

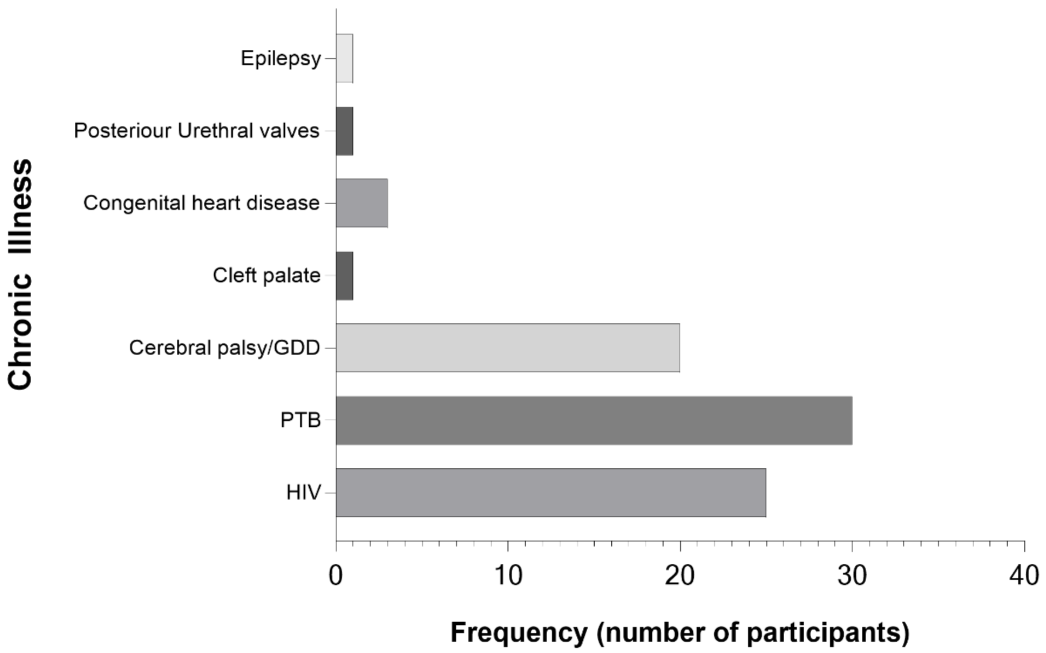

Figure S1. Frequency of chronic illnesses among children hospitalized with acute malnutrition. HIV; Human immunodeficiency virus, TB; Tuberculosis, GDD; Global developmental delay.

Supplementary Table S1: Comparison of participants characteristics with single value vs. serial creatinine values

	Single creatinine (n=18)	Serial creatinine values (n=167)	p-Value
**Clinical characteristics**			
Age, years, median (IQR)	1.1 (0.8, 1.5)	1.2 (0.9, 1.7)	0.385
Sex, n (%) Female	6 (33.3)	61 (36.5)	0.789
Fever, n (%)	6 (33.3)	57 (34.1)	0.946
Features of fluid loss			
No diarrhea/ vomiting	4 (22.2)	51 (30.5)	0.167
Either diarrhea/ vomiting	3 (16.7)	54 (32.3)	
Both diarrhea & vomiting	11 (61.1)	62 (37.1)	
Unable to drink/breastfeed, n (%)	1 (5.6)	34 (20.4)	0.204
Anemia	12 (66.7)	108 (64.7)	0.866
Reduce urine output, n (%)	4 (22.2)	5 (3.0)	0.006
AKI risk score	1.0 (1.0, 3.0)	2.0 (1.0, 3.0)	0.876
**Comorbid conditions**			
Cerebral palsy or GDD	0 (0.0)	20 (12.0)	0.226
HIV infection, n (%)	2 (11.1)	23 (13.8)	1.000
TB, n (%)	2 (11.1)	28 (16.8)	0.742
Sepsis, n (%)	7 (38.9)	60 (35.9)	0.804
Malaria, n (%)	0 (0.0)	9 (5.4)	0.603
**Medications**			
Paracetamol	1 (5.6)	40 (24.0)	0.130
Ampicillin	15 (83.3)	131 (78.4)	0.629
Amikacin	16 (88.9)	142 (85.0)	0.660
Ceftriaxone	5 (27.8)	108 (64.7)	0.002
HAART, n (%)	1 (5.6)	18 (10.8)	0.699
Anti-TB medications	2 (11.1)	28 (16.8)	0.742
**Laboratory Findings**			
WBC x 10^3/μL	11.9 (8.2, 16.3)	11.8 (8.7, 16.1)	0.931
Neutrophil count x 10^3/μL	3.8 (1.7, 9.8)	3.8 (2.3, 6.8)	0.830
Lymphocyte count x 10^3/μL	4.4 (2.9, 6.3)	5.3 (3.8, 7.9)	0.071
Hemoglobin, g/dL	9.9 (7.9, 10.9)	9.8 (8.3, 11.1)	0.944
Platelet count x10^3/μL	349.6 (149.0, 519.9)	393.0 (262.0, 517.0)	0.236
Admission creatinine	0.4 (0.3, 1.0)	0.2 (0.2, 0.4)	0.001
Sodium (mmol/L)	137.0 (133.0, 139.0)	135.2 (133.0, 138.0)	0.249
Potassium (mmol/L)	3.8 (3.6, 4.1)	3.9 (3.6, 4.2)	0.990
Chloride (mmol/L)	97.9 (96.0, 98.0)	98.0 (96.0, 99.0)	0.373

p-values generated using Chi-square or Fishers T- test as appropriate for categorical variables and using the Wilcoxon rank sum test for continuous variables. Abbreviations: HAART, highly active antiretroviral therapy; HIV – human immunodeficiency virus, IQR, interquartile range; SCr, serum creatinine; TB, Tuberculosis; WBC – white blood cells
† statistically significant p-value <0.05

Supplementary Table S2: Sensitivity and specificity of different AKI definitions to predict mortality

	Sensitivity % (95% CI)	Specificity (95% CI)	Positive predictive value (95% CI)	Negative predictive value (95% CI)
**AKI definition**				
AKI_{all}	56.0 (48.9, 63.2)	56.3 (49.1, 63.4)	16.7 (11.3, 22.0)	89.1 (84.6, 93.6)
$AKI_{0.4}$	52.0 (44.8, 59.2)	81.3 (75.6, 86.9)	30.2 (23.6, 36.9)	91.6 (87.5, 95.6)
$AKI_{0.5}$	52.0 (44.8, 59.2)	91.9 (87.9, 95.8)	50.0 (42.8, 57.2)	92.5 (88.7, 96.3)

Supplementary Table S3: Comparison of participants characteristics across different AKI definitions (modifying absolute creatinine thresholds)

	No SCr threshold		SCr 0.4 mg/dL threshold		SCr 0.5 mg/dL threshold	
	**No AKI (n=101)**	**AKI (n= 84)**	**No AKI (n=142)**	**AKI (n=43)**	**No AKI (n=159)**	**AKI (n= 26)**
**Clinical characteristics**						
Age, years, median (IQR)	1.2 (0.9, 1.7)	1.1 (0.8, 1.7)	1.2 (0.9, 1.7)	1.1 (0.7, 2.1)	1.2 (0.9, 1.7)	0.9 (0.6, 1.7)
Sex, n (%) Female	37 (36.6)	30 (35.7)	50 (35.2)	17 (39.5)	57 (35.8)	10 (38.5)
Fever, n (%)	39 (38.6)	24 (28.6)	47 (33.1)	16 (37.2)	50 (31.4)	13 (50.0)
Features of fluid loss						
No diarrhea/ vomiting	37 (36.6)	18 (21.4)	**49 (34.5)**	**6 (14.0) †**	**52 (32.7)**	**3 (11.5) †**
Either diarrhea/ vomiting	29 (28.7)	28 (33.3)	**44 (31.0)**	**13 (30.2)**	**49 (30.8)**	**8 (30.8)**
Both diarrhea & vomiting	35 (34.7)	38 (45.2)	**49 (34.5)**	**24 (55.8)**	**58 (36.5)**	**15 (57.7)**
Unable to drink/breastfeed, n (%)	19 (18.8)	16 (19.0)	24 (16.9)	11 (25.6)	31 (19.5)	4 (15.4)
Anemia	69 (68.3)	51 (60.7)	95 (66.9)	25 (58.1)	105 (66.0)	15 (57.7)
Reduce urine output, n (%)	5 (5.0)	4 (4.8)	6 (4.2)	3 (7.0)	7 (4.4)	2 (7.7)
AKI risk score	2 (1,3)	2 (1,3)	2 (1,3)	2 (1,3)	2 (1,3)	2 (1,3)
Length of hospitalization (days)	16 (9, 24)	16 (12, 22)	16 (9, 24)	16 (13, 22)	15 (9, 24)	21 (14, 25)
**Comorbid conditions**						
HIV infection, n (%)	13 (12.9)	12 (14.3)	18 (12.7)	7 (16.3)	19 (11.9)	6 (23.1)
TB, n (%)	23 (22.8)	7 (8.3) †	24 (16.9)	6 (14.0)	24 (15.1)	6 (23.1)
Sepsis, n (%)	36 (35.6)	31 (36.9)	46 (32.4)	21 (48.8)	54 (34.0)	13 (50.0)
Malaria, n (%)	5 (5.0)	4 (4.8)	9 (6.3)	0 (0.0)	9 (5.7)	0 (0.0)
**Medications**						
Paracetamol	18 (17.8)	23 (27.4)	28 (19.7)	13 (30.2)	33 (20.8)	8 (30.8)
Ampicillin	81 (80.2)	65 (77.4)	115 (81.0)	31 (72.1)	125 (78.6)	21 (80.8)
Amikacin	84 (83.2)	74 (88.1)	121 (85.2)	37 (86.0)	134 (84.3)	24 (92.3)
Ceftriaxone	60 (59.4)	53 (63.1)	84 (59.2)	29 (67.4)	96 (60.4)	17 (65.4)
HAART, n (%)	10 (9.9)	9 (10.7)	14 (9.9)	5 (11.6)	15 (9.4)	4 (15.4)
Anti-TB medications	23 (22.8)	7 (8.3) †	24 (16.9)	6 (14.0)	24 (15.1)	6 (23.1)
**Laboratory Findings**						
WBC x 10^3/μL	11.1 (8.6, 15.7)	12.7 (8.9, 16.5)	**11.1 (8.6, 14.9)**	**14.2 (8.6, 20.8)†**	**11.1 (8.6, 15.7)**	**15.5 (11.9, 27.4)†**
Neutrophil count x 10^3/μL	3.5 (2.2, 5.3)	4.1 (2.4, 8.8)	**3.4 (2.2, 5.2)**	**6.0 (2.7, 12.2)†**	**3.5 (2.2, 5.5)**	**8.4 (3.6, 17.2)†**
Lymphocyte count x 10^3/μL	5.4 (3.7, 7.5)	5.1 (3.6, 8.3)	5.4 (3.9, 7.7)	5.0 (3.2, 7.9)	5.2 (3.9, 7.7)	4.7 (2.5, 7.9)
Hemoglobin, g/dL	9.8 (8.4, 11.1)	10.0 (8.0, 11.0)	9.9 (8.4, 11.1)	9.6 (7.8, 11.1)	9.8 (8.4, 11.1)	9.8 (7.8, 10.9)
Platelet count x10^3/μL	394 (274, 520)	365 (238, 516)	388 (269, 520)	408 (228, 514)	387 (258, 520)	412 (228, 472)
Sodium (mmol/L)	135 (134, 138)	135 (133, 139)	135 (133, 138)	136 (133, 139)	135 (133, 138)	135 (133, 139)
Potassium (mmol/L)	3.8 (3.7, 4.1)	3.9 (3.6, 4.3)	3.8 (3.6, 4.1)	3.9 (3.7, 4.3)	3.9 (3.6, 4.2)	3.9 (3.6, 4.6)
Chloride (mmol/L)	98 (96, 99)	98 (96, 100)	98 (96, 99)	98 (96, 99)	98 (96, 99)	97 (95, 99)

p-values generated using Chi-square or Fishers T- test as appropriate for categorical variables and using the Wilcoxon rank sum test for continuous variables.
Abbreviations: HAART, highly active antiretroviral therapy; HIV – human immunodeficiency virus, IQR, interquartile range; SCr, serum creatinine; TB, Tuberculosis; WBC – white blood cells

† statistically significant p-value <0.05

Table S4: Sex disaggregated presentation of clinical characteristics, AKI, and mortality

	**Male (n=118)**	**Female (n=67)**	**p-Value**
**Clinical characteristics**			
Age, years, median (IQR)	1.2 (0.9, 1.8)	1.1 (0.9, 1.7)	0.373
Fever, n (%)	45 (38.1)	18 (26.9)	0.120
Features of fluid loss			
No diarrhea/ vomiting	31 (26.3)	24 (35.8)	0.375
Either diarrhea/ vomiting	39 (33.1)	18 (26.9)	
Both diarrhea & vomiting	48 (40.7)	25 (37.3)	
Unable to drink/breastfeed, n (%)	19 (16.1)	16 (23.9)	0.194
Anemia	78 (66.1)	42 (62.7)	0.640
Reduce urine output, n (%)	6 (5.1)	3 (4.5)	1.000
**Comorbid conditions**			
HIV infection, n (%)	13 (11.0)	12 (17.9)	0.187
Tuberculosis, n (%)	20 (16.9)	10 (14.9)	0.720
Sepsis, n (%)	43 (36.4)	24 (35.8)	0.933
Malaria, n (%)	5 (4.2)	4 (6.0)	0.725
**Medications during hospitalization**			
Paracetamol	24 (20.3)	17 (25.4)	0.428
Ampicillin	95 (80.5)	51 (76.1)	0.482
Amikacin	103 (87.3)	55 (82.1)	0.336
Ceftriaxone	72 (61.0)	41 (61.2)	0.981
HAART, n (%)	9 (7.6)	10 (14.9)	0.116
Anti TB medications	20 (16.9)	10 (14.9)	0.720
**Laboratory findings**			
WBC x $10^3/\mu L$	11.3 (8.3, 15.7)	12.5 (8.9, 17.7)	0.149
Neutrophil count x $10^3/\mu L$	3.9 (2.3, 7.3)	3.8 (2.2, 6.0)	0.968
Lymphocyte count x $10^3/\mu L$	5.1 (3.6, 7.3)	6.0 (3.9, 8.4)	0.077
Hemoglobin, g/dL	9.7 (8.3, 10.9)	10.1 (8.3, 11.2)	0.428
Platelet count x10^9/L	386 (258, 520)	398 (237, 513)	0.955
Sodium (mmol/L)	136 (133, 138)	135 (133, 138)	0.326
Potassium (mmol/L)	3.9 (3.6, 4.2)	3.8 (3.6, 4.1)	0.344
Chloride (mmol/L)	98.0 (96.0, 99.0)	98.0 (95.3, 99.0)	0.264
AKI_{all} (no creatinine threshold)	54 (45.8)	30 (44.8)	0.897
$AKI_{0.4}$ (creatinine threshold 0.4mg/dL)	26 (22.0)	17 (25.4)	0.605
$AKI_{0.5}$ (creatinine threshold 0.5mg/dL)	16 (13.6)	10 (14.9)	0.797
In-hospital mortality, n (%)	15 (12.7)	10 (14.9)	0.672

P-values generated using Chi-square or Fishers exact test as appropriate for categorical variables and using the Wilcoxon rank sum test for continuous variables.

STROBE Statement—checklist of items that should be included in reports of observational studies

	Item No	Recommendation	Page No
**Title and abstract**	1	(*a*) Indicate the study's design with a commonly used term in the title or the abstract	Provided in the abstract methods section
		(*b*) Provide in the abstract an informative and balanced summary of what was done and what was found	Provided in the abstract
**Introduction**			
Background/rationale	2	Explain the scientific background and rationale for the investigation being reported	Explained in the background section
Objectives	3	State specific objectives, including any prespecified hypotheses	Stated in the last paragraph of the background section
**Methods**			
Study design	4	Present key elements of study design early in the paper	Presented in the first paragraphs of methods section under the sub-section study design
Setting	5	Describe the setting, locations, and relevant dates, including periods of recruitment, exposure, follow-up, and data collection	Described in the methods section
Participants	6	(*a*) *Cohort study*—Give the eligibility criteria, and the sources and methods of selection of participants. Describe methods of follow-up	Described in the methods section
Variables	7	Clearly define all outcomes, exposures, predictors, potential confounders, and effect modifiers. Give diagnostic criteria, if applicable	Variables including diagnostic criteria described in the methods section and in the supplementary methods
Data sources/ measurement	8*	For each variable of interest, give sources of data and details of methods of assessment (measurement). Describe comparability of assessment methods if there is more than one group	The data sources and measurements are in the methods section
Bias	9	Describe any efforts to address potential sources of bias	This is addressed in data analysis section under the methods section
Study size	10	Explain how the study size was arrived at	This is provided in the methods section in the last paragraph of the study design sub-section

Quantitative variables	11	Explain how quantitative variables were handled in the analyses. If applicable, describe which groupings were chosen and why	Explained in the data analysis section under the methods section
Statistical methods	12	(*a*) Describe all statistical methods, including those used to control for confounding	Explained in the data analysis section under the methods section
		(*b*) Describe any methods used to examine subgroups and interactions	Explained in the data analysis section under the methods section
		(*c*) Explain how missing data were addressed	Explained in the data analysis section and the methods section
		(*d*) *Cohort study*—If applicable, explain how loss to follow-up was addressed	Explained in the methods section
		(*e*) Describe any sensitivity analyses	Explained in the data analysis section under the methods section

Continued on next page

Results

Participants	13*	(a) Report numbers of individuals at each stage of study—eg numbers potentially eligible, examined for eligibility, confirmed eligible, included in the study, completing follow-up, and analysed	Paragraph one of the results section (description of population sub-section) and Figure 1; Flow chart of the study population
		(b) Give reasons for non-participation at each stage	
		(c) Consider use of a flow diagram	
Descriptive data	14*	(a) Give characteristics of study participants (eg demographic, clinical, social) and information on exposures and potential confounders	Results section paragraph 1, 2 and 3. The sub-sections are; Description of study population, Defining AKI and the association with mortality, and Clinical parameters associated with AKI. This information is provided in Table 1 and Supplementary Table S1.
		(b) Indicate number of participants with missing data for each variable of interest	
		(c) *Cohort study*—Summarise follow-up time (eg, average and total amount)	
Outcome data	15*	*Cohort study*—Report numbers of outcome events or summary measures over time	Paragraph 2 and 3 of the results section. These are under sub-sections are: Description of study population, Defining AKI and the association with mortality, and Clinical parameters associated with AKI. Figure 1, and Supplementary Table S3
Main results	16	(*a*) Give unadjusted estimates and, if applicable, confounder-adjusted estimates and their precision (eg, 95% confidence interval). Make clear which confounders were adjusted for and why they were included	Results section paragraph 2 and 3; under the sub-sections defining AKI and the association with mortality, and clinical parameters. Table 2, Figure 2 and Supplementary Table S2
		(*b*) Report category boundaries when continuous variables were categorized	
		(*c*) If relevant, consider translating estimates of relative risk into absolute risk for a meaningful time period	
Other analyses	17	Report other analyses done—eg analyses of subgroups and interactions, and sensitivity analyses	Results section paragraph 4 sub-section 'comparison of risk scores in predicting AKI; and Table 3 and Figure 3

Discussion

Key results	18	Summarise key results with reference to study objectives	Paragraph 1 of the discussion section

Limitations	19	Discuss limitations of the study, taking into account sources of potential bias or imprecision. Discuss both direction and magnitude of any potential bias	Paragraph 7 of the discussion section
Interpretation	20	Give a cautious overall interpretation of results considering objectives, limitations, multiplicity of analyses, results from similar studies, and other relevant evidence	Discussion section and conclusion
Generalisability	21	Discuss the generalisability (external validity) of the study results	Discussion section
**Other information**			
Funding	22	Give the source of funding and the role of the funders for the present study and, if applicable, for the original study on which the present article is based	Under section of Funding
